# Using landscape habitat associations to prioritize areas of conservation action for terrestrial birds

**DOI:** 10.1371/journal.pone.0173041

**Published:** 2017-03-16

**Authors:** Tyler M. Harms, Kevin T. Murphy, Xiaodan Lyu, Shane S. Patterson, Karen E. Kinkead, Stephen J. Dinsmore, Paul W. Frese

**Affiliations:** 1 Center for Survey Statistics and Methodology, Iowa State University, 208 Office and Laboratory Building, 2401 Osborn Drive, Ames, Iowa, United States of America; 2 Department of Natural Resource Ecology and Management, Iowa State University, 339 Science Hall II, 2310 Pammel Drive, Ames, Iowa, United States of America; 3 Department of Statistics, Iowa State University, 1121 Snedecor Hall, 2438 Osborn Drive, Ames, Iowa, United States of America; 4 Iowa Department of Natural Resources, Wildlife Diversity Program, Boone, Iowa, United States of America; Università degli Studi di Napoli Federico II, ITALY

## Abstract

Predicting species distributions has long been a valuable tool to plan and focus efforts for biodiversity conservation, particularly because such an approach allows researchers and managers to evaluate species distribution changes in response to various threats. Utilizing data from a long-term monitoring program and land cover data sets, we modeled the probability of occupancy and colonization for 38 bird Species of Greatest Conservation Need (SGCN) in the robust design occupancy modeling framework, and used results from the best models to predict occupancy and colonization on the Iowa landscape. Bird surveys were conducted at 292 properties from April to October, 2006–2014. We calculated landscape habitat characteristics at multiple spatial scales surrounding each of our surveyed properties to be used in our models and then used kriging in ArcGIS to create predictive maps of species distributions. We validated models with data from 2013 using the area under the receiver operating characteristic curve (AUC). Probability of occupancy ranged from 0.001 (SE < 0.001) to 0.995 (SE = 0.004) for all species and probability of colonization ranged from 0.001 (SE < 0.001) to 0.999 (SE < 0.001) for all species. AUC values for predictive models ranged from 0.525–0.924 for all species, with 17 species having predictive models considered useful (AUC > 0.70). The most important predictor for occupancy of grassland birds was percentage of the landscape in grassland habitat, and the most important predictor for woodland birds was percentage of the landscape in woodland habitat. This emphasizes the need for managers to restore specific habitats on the landscape. In an era during which funding continues to decrease for conservation agencies, our approach aids in determining where to focus limited resources to best conserve bird species of conservation concern.

## Introduction

Research on the conservation of biodiversity has become increasingly important in the last two decades, particularly in the face of threats such as habitat loss and fragmentation [1–3], climate change [3–5], invasive species [6,7], and many others. Humans are responsible for several threats to wildlife, primarily habitat loss. As the human population continues to grow and human needs increase, many animals will continue to suffer due to habitat loss. Of all the biodiversity “hotspots” remaining in the world, only one-third of the historic habitat supporting the high biodiversity in these areas remains [1]. Although habitat loss and degradation affects all wildlife, it has drastic effects on birds. Nearly 85% of the globally threatened bird species [8] are significantly threatened by habitat loss. Such effects on birds are also evident at localized scales, for example Iowa has lost 57% of historic forest habitat, 95% of historic wetland habitat, and 99.9% of historic grassland habitat since European settlement [9]. As a result, nearly 30% of Iowa’s breeding and migratory birds are considered Species of Greatest Conservation Need (SGCN), and a majority of these species are also of heightened conservation status in the Midwest United States [10]. Funding for the conservation of biodiversity and habitat management is severely lacking [11,12] despite the increasing threats mentioned above. Therefore, identification of priority areas (i.e., areas where the most species can be benefitted with the least amount of cost) is critical to effective conservation planning [11,13].

In 2003, Congress asked all U.S. states to develop a proactive plan to assess the status of wildlife populations, to identify potential issues facing wildlife in the future, outline and prioritize actions to conserve all wildlife populations in perpetuity, and identify species in need of conservation action (e.g., SGCN). Known as State Wildlife Action Plans (SWAPs), they were required of states in order to receive funding through the State and Tribal Wildlife Grants Program, and by 2005 all 50 states had developed a SWAP [14]. In response to these plans, some states including Iowa launched large-scale inventory and monitoring efforts to evaluate the status of wildlife populations within their borders, inform conservation actions, and continue monitoring wildlife populations as a response to habitat restoration and management and a changing landscape (Iowa Multiple Species Inventory and Monitoring (MSIM) Program; http://www.iowadnr.gov/Environment/WildlifeStewardship/NonGameWildlife/DiversityProjects/MSIM.aspx). Data collected through these monitoring efforts can be used in models to evaluate habitat associations of all wildlife species, particular those SGCN, and to identify priority areas for conservation action or areas of high biodiversity based on predicted occupancy of SGCN. These proactive approaches for prioritizing areas of conservation action can help reduce the impact of habitat loss and alteration on wildlife, thus maintaining biodiversity.

Predicting the distribution of species of conservation concern has long been considered a valuable tool for conservation planning [15,16] and for the conservation of biodiversity [17]. The benefits of these tools are numerous, allowing biologists and land managers the opportunity to evaluate how species will respond to habitat characteristics on the landscape in order to focus habitat restoration and management efforts [18–20], and how species will respond to different climate scenarios in the face of global change [15,21–23]. Even more valuable is the coupling of data from long-term monitoring projects, such as those mentioned above, with predictive modeling efforts to evaluate spatial and temporal trends in species distributions [17,24]. Natural resource agencies are continually faced with decisions to prioritize conservation actions based on limited funding, and monitoring and species distribution models can provide scientific information to aid in prioritization.

In this study, we utilized robust design occupancy models [25] to evaluate landscape-scale habitat associations of 38 terrestrial bird SGCN in Iowa using data collected through the Iowa MSIM Program developed under the Iowa SWAP [9]. We then developed a spatially-explicit prediction of the probability of occupancy of each species across Iowa using results from the above models. Using occupancy models to predict occupancy of species is a preferred method because such models incorporate the probability of detecting a given species when estimating the probability of occupancy, thus minimizing the risk of under-predicting occupancy and increasing predictive performance [17,26]. Our overall objective was to develop an approach for predicting species occupancy and colonization using long-term monitoring data and landscape characteristics with robust design occupancy models. We then applied our approach to develop maps identifying priority areas for targeted conservation action for SGCN birds, which could later be combined to facilitate multi-species conservation and increase biodiversity conservation in Iowa.

## Materials and methods

### Site selection and survey point establishment

**Fig 1:**
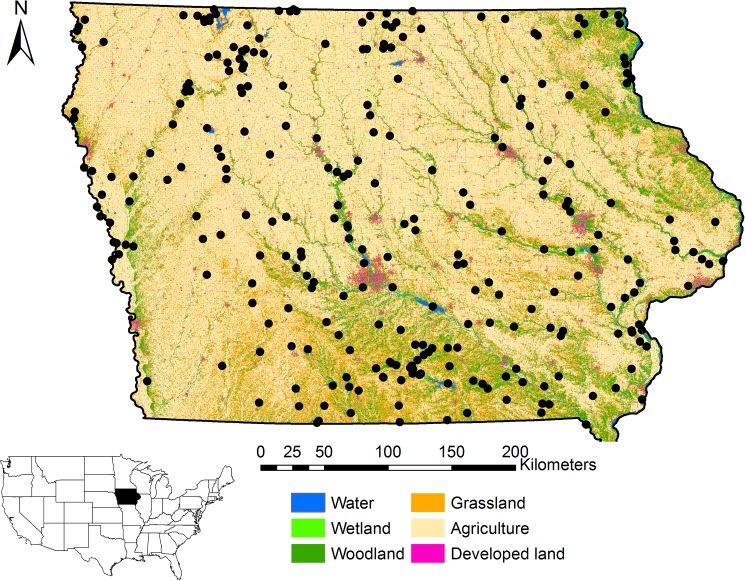
All sites surveyed for birds (black dots) as part of the Iowa Multiple Species Inventory and Monitoring (MSIM) Program in Iowa, 2006–2014.

Our work encompassed a wide range of terrestrial and aquatic habitats throughout Iowa. We selected sites to be surveyed for birds using a stratified random sampling design (Fig 1). All public properties in Iowa >98 ha (approximately 250 ac) were classified according to 19 habitat types outlined in the Iowa SWAP [9]. We considered only public properties for ease of access. In addition, we considered only those public properties >98 ha to reduce our sampling frame due to financial and logistical constraints. We stratified properties into quarters of the state by splitting the state in half along both north-south and east-west gradients to allow for equal selection of different habitat types across the state. We selected new properties without replacement each year from 2006–2014 such that properties of a certain habitat type were selected from each management district. We also retained 1–5 properties from the sample of properties each year to constitute a sample of properties surveyed multiple years for comparison purposes. By 2014, this resulted in 26 properties being surveyed annually. No specific permission was needed to collect data on properties owned by the Iowa Department of Natural Resources or various County Conservation Boards. Permission and Special Use Permits were obtained from the U.S. Fish and Wildlife Service for data collection on National Wildlife Refuges (e.g., DeSoto Bend National Wildlife Refuge, Union Slough National Wildlife Refuge, Upper Mississippi National Fish and Wildlife Refuge). Permission was obtained from the National Park Service for data collection on Effigy Mounds National Monument. Our study did not include data collection for any threatened or endangered species. Field methods for this study were reviewed and approved by the Iowa State University Institutional Animal Care and Use Committee (IACUC; Protocol #3-12-7326-Q).

We established a core survey area on each property that encompassed the assigned habitat type of that property. Core areas on each property were identified as the area of the property that contained the largest contiguous patch of particular habitat type assigned to that property. Within the core area, we established seven points approximately 200 m apart and in a hexagonal shape (including one point in the center) to allow for adequate coverage of the core area while minimizing double counting birds [27]. Surveys were only conducted within the core habitat area on each property.

### Bird surveys

We conducted bird surveys at selected properties from April–October each year from 2006–2014. We divided the survey year into three seasons to focus on both breeding and migratory birds: spring (April–May) and fall (September–October) focused on migratory birds and summer (June–July) focused on breeding birds. We conducted three visits to each property at least 4 d apart in each of the three seasons for a total of 9 visits to each property in a survey year. On each visit, we conducted standardized, 10-min point counts with distance sampling at all seven survey points from 30 m before sunrise to 4 hr after sunrise. We recorded all birds seen or heard at each point, estimated the linear distance to each bird seen or heard, and placed the bird into one of five distance categories: 0–25 m, 26–50 m, 51–75 m, 76–100 m, and >101 m. We considered the site occupied if a species of interest was detected during at least one of the seven point counts. Adhering to the primary assumption of distance sampling [28], we recorded the distance to each individual bird when it was first observed and did not record any subsequent observations. Prior to bird surveys, we measured wind speed (km/h), cloud cover (%), and temperature (°C) and did not conduct bird surveys during periods of fog, prolonged precipitation, or high winds (>20 km/h).

### Landscape habitat covariates

Using ArcGIS [ver. 10.1; 29], we measured various landscape-level habitat variables within a 200 m, 500 m, and 1000 m radius of each sampled site. We placed a buffer around each of our sampled sites using the buffer tool in ArcGIS toolbox [Analysis Tools, Proximity, Buffer; 29]. Next, we clipped the 2009 Iowa Landcover file to our site buffers using the “clipraster” command in the tools extension package Geospatial Modeling Environment [GME; 30]. The 2009 Iowa Landcover file provides information on the land use classification of the Iowa landscape in 2009 using satellite imagery at a 3 m resolution and includes classifications such as “grassland”, “forest”, and “wetland” among others [31]. This is currently the most recent land use classification for Iowa. We repeated the above two steps for both radii to obtain the land use description within each radius our surveyed sites. Among the various land-use classifications in the Landcover file, we selected the “water”, “wetland”, “grassland”, “woodland”, and “agriculture” classifications for our analysis because these were the classifications we believed would most influence our focal species [32,33].

We estimated our landscape-level habitat characteristics at each scale using FRAGSTATS [ver. 4.2; 34]. For our analyses, we selected the percentage of landscape (PLAND), largest patch index (LPI), edge density (ED), patch density (PD), and interspersion-juxtaposition (IJI) metrics. Percentage of landscape measures the area of the focal land-use classification standardized by the total area of the landscape. LPI is the largest patch of the corresponding land-use classification standardized by the total landscape area. ED measures the amount of edge on the landscape corresponding to a single land-use classification standardized by landscape area. PD measures the number of patches on the landscape corresponding to a single land-use classification standardized by area. Lastly, IJI measures the degree to which patches of different land-use classifications are interspersed among each other based on patch adjacencies. We performed these four calculations on the five land-use classifications for each scale resulted in 75 landscape-level variables to be included as covariates in our models (Table 1). We then assessed correlation among our habitat variables using a simple correlation matrix. Highly correlated combinations of two variables (R>0.60 or R<-0.60; n = 129) were not included in the same model.

**Table 1:** Final list of landscape-level habitat covariates modeled on probability of occupancy and colonization.

Land-use classification	Spatial scale	Variable name
Agriculture	200 m	Edge density
Agriculture	200 m	Interspersion-juxtaposition
Agriculture	200 m	Largest patch index
Agriculture	200 m	Percentage of landscape
Agriculture	200 m	Patch density
Agriculture	500 m	Edge density
Agriculture	500 m	Interspersion-juxtaposition
Agriculture	500 m	Largest patch index
Agriculture	500 m	Percentage of landscape
Agriculture	500 m	Patch density
Agriculture	1000 m	Edge density
Agriculture	1000 m	Interspersion-juxtaposition
Agriculture	1000 m	Largest patch index
Agriculture	1000 m	Percentage of landscape
Agriculture	1000 m	Patch density
Grassland	200 m	Edge density
Grassland	200 m	Interspersion-juxtaposition
Grassland	200 m	Largest patch index
Grassland	200 m	Percentage of landscape
Grassland	200 m	Patch density
Grassland	500 m	Edge density
Grassland	500 m	Interspersion-juxtaposition
Grassland	500 m	Largest patch index
Grassland	500 m	Percentage of landscape
Grassland	500 m	Patch density
Grassland	1000 m	Edge density
Grassland	1000 m	Interspersion-juxtaposition
Grassland	1000 m	Largest patch index
Grassland	1000 m	Percentage of landscape
Grassland	1000 m	Patch density
Woodland	200 m	Edge density
Woodland	200 m	Interspersion-juxtaposition
Woodland	200 m	Largest patch index
Woodland	200 m	Percentage of landscape
Woodland	200 m	Patch density
Woodland	500 m	Edge density
Woodland	500 m	Interspersion-juxtaposition
Woodland	500 m	Largest patch index
Woodland	500 m	Percentage of landscape
Woodland	500 m	Patch density
Woodland	1000 m	Edge density
Woodland	1000 m	Interspersion-juxtaposition
Woodland	1000 m	Largest patch index
Woodland	1000 m	Percentage of landscape
Woodland	1000 m	Patch density
Wetland	200 m	Edge density
Wetland	200 m	Interspersion-juxtaposition
Wetland	200 m	Largest patch index
Wetland	200 m	Percentage of landscape
Wetland	200 m	Patch density
Wetland	500 m	Edge density
Wetland	500 m	Interspersion-juxtaposition
Wetland	500 m	Largest patch index
Wetland	500 m	Percentage of landscape
Wetland	500 m	Patch density
Wetland	1000 m	Edge density
Wetland	1000 m	Interspersion-juxtaposition
Wetland	1000 m	Largest patch index
Wetland	1000 m	Percentage of landscape
Wetland	1000 m	Patch density
Water	200 m	Edge density
Water	200 m	Interspersion-juxtaposition
Water	200 m	Largest patch index
Water	200 m	Percentage of landscape
Water	200 m	Patch density
Water	500 m	Edge density
Water	500 m	Interspersion-juxtaposition
Water	500 m	Largest patch index
Water	500 m	Percentage of landscape
Water	500 m	Patch density
Water	1000 m	Edge density
Water	1000 m	Interspersion-juxtaposition
Water	1000 m	Largest patch index
Water	1000 m	Percentage of landscape
Water	1000 m	Patch density

### Robust design occupancy models

We utilized the robust design occupancy model framework [25] in Program Mark [35] to evaluate the effects of the above-mentioned landscape-level habitat characteristics on terrestrial birds in Iowa. The robust design occupancy model estimates four parameters: 1) probability of occupancy (ψ), or the probability that the species of interest occupied a sampled site, 2) probability of colonization (γ), or the probability that a site will was colonized at time *t*+1 given the site was not occupied at time *t*, 3) probability of extinction (ε), or the probability that a site went extinct at time *t*+1 given the site was occupied at time *t*, and 4) detection probability, or the probability of detecting the species of interest given it was present at the sampled site [*p*; 25]. For all species, we utilized the reduced robust design occupancy model that estimates ψ, γ, and *p* for our analyses for two reasons: 1) we were more interested in estimates of γ because it provides information on potential habitats to restore to benefit SGCN birds and 2) models were more likely to converge due to parsimony. Unlike the single-season occupancy model where sites are closed to changes in occupancy state during the primary sampling season [36], the robust design occupancy model assumes sites are closed to changes in occupancy state between secondary sampling intervals (e.g., sampling occasions within a year) but are open to changes in occupancy state between primary sampling intervals [e.g., years; 25]. This allows for the evaluation of meta-population dynamics through the process of determining the probability a site will remain occupied, go locally extinct, or become locally colonized. In addition, the robust design occupancy model allows covariates to be modeled on the parameters to improve parameter estimates and provide information on characteristics might influence the various parameters.

We modeled landscape-level habitat variables on probability of occupancy and probability of colonization for 38 species of terrestrial birds (Table 2) listed as SGCN by the Iowa Wildlife Action Plan [9]. We divided the species list into four guilds based on primary habitat associations: grassland, woodland, scrub-shrub, and all other species (Table 2). The primary sampling intervals were the years during which bird surveys were conducted (2006–2014) and the secondary sampling intervals were the survey occasions (days) with each sampling year (April-October). For each guild, we modeled the same set of habitat variables and interactions for all scales on both occupancy and colonization probabilities based on biological knowledge and review of the literature. For example, we modeled all grassland, woodland, and agriculture variables for all scales on birds within the grassland guild as well as two-way interactions of all grassland and woodland and grassland and agriculture variables. We also modeled time-varying covariates of wind speed, cloud cover, and temperature on detection probability. We estimated all parameters on an annual basis. We truncated data sets to the known breeding season for each species [37] to ensure closure among the secondary sampling occasions. For species that do not breed statewide (7 of 38 species), we restricted data sets by landform region [38] to surveyed sites within core breeding areas as determined by Iowa Breeding Bird Atlas data [39]. We did not consider migratory species because they violate the closure assumption of occupancy models [36]. Because we had landcover data from only one year (2009), we assumed the landscape and the corresponding effects on occupancy and colonization did not change among our survey years and pooled all survey years for analysis. We evaluated models using Akaike’s Information Criterion adjusted for small sample sizes [AIC_c_; 40]. Models with ΔAIC_c_≤2 were considered to have strong support [40].

**Table 2:** List of species, their respective guild, and estimates (standard error; SE) for occupancy (Psi), colonization (Gamma), and detection (p) probabilities, and area under the receiver operating characteristic curve (AUC).

Species	Guild	ψ (SE)	γ (SE)	p (SE)	AUC
Trumpeter Swan (*Cygnus buccinator*)	Other	0.071 (0.009)	0.043 (0.005)	0.247 (0.040)	0.673
Northern Bobwhite (*Colinus virginianus*)	Scrub-shrub	0.256 (0.009)	0.145 (0.007)	0.540 (0.042)	0.697
American Bittern (*Botaurus lentiginosis*)	Other	0.193 (0.075)	NE	0.099 (0.038)	0.600
Bald Eagle (*Haliaeetus leucocephalus*)	Other	0.277 (0.066)	0.039 (0.038)	0.140 (0.024)	0.541
Northern Harrier (*Circus cyaneus*)	Grassland	NE	0.275 (0.016)	0.775 (0.112)	NE
Red-shouldered Hawk (*Buteo lineatus*)	Woodland	0.132 (0.016)	0.073 (0.009)	0.267 (0.044)	0.798
Broad-winged Hawk (*Buteo platypterus*)	Woodland	NE	NE	NE	NE
Upland Sandpiper (*Bartramia longicauda*)	Grassland	NE	0.023 (0.011)	0.368 (0.085)	NE
American Woodcock (*Scolopax minor*)	Other	NE	NE	NE	NE
Yellow-billed Cuckoo (*Coccyzus americanus*)	Woodland	0.668 (0.041)	0.387 (0.042)	0.532 (0.025)	0.706
Black-billed Cuckoo (*Coccyzus erythropthalmus*)	Woodland	0.576 (0.254)	0.037 (0.032)	0.120 (0.029)	0.743
Common Nighthawk (*Chordeiles minor*)	Other	NE	NE	NE	NE
Belted Kingfisher (*Megaceryle alcyon*)	Other	0.505 (0.052)	0.333 (0.104)	0.197 (0.019)	0.640
Red-headed Woodpecker (*Melanerpes erythrocephalus*)	Woodland	0.572 (0.030)	0.276 (0.061)	0.622 (0.024)	0.610
Northern Flicker (*Colaptes auratus*)	Woodland	0.900 (0.029)	NE	0.600 (0.019)	0.656
American Kestrel (*Falco sparverius*)	Grassland	0.194 (0.008)	0.999 (<0.001)	0.055 (0.008)	0.793
Eastern Wood-Pewee (*Contopus virens*)	Woodland	0.979 (0.001)	0.481 (0.008)	0.861 (0.013)	0.906
Acadian Flycatcher (*Empidonax virescens*)	Woodland	0.031 (0.002)	0.068 (0.004)	0.584 (0.040)	0.892
Eastern Kingbird (*Tyrannus tyrannus*)	Grassland	0.762 (0.002)	0.101 (0.002)	0.581 (0.016)	0.722
Bell's Vireo (*Vireo bellii*)	Scrub-shrub	0.053 (0.002)	0.056 (0.004)	0.336 (0.067)	0.732
Horned Lark (*Eremophilia alpestris*)	Grassland	NE	NE	NE	NE
Bank Swallow (*Riparia riparia*)	Other	0.328 (0.020)	0.203 (0.011)	0.202 (0.023)	0.606
Sedge Wren (*Cisthorus platensis*)	Grassland	0.433 (0.009)	0.388 (0.031)	0.621 (0.022)	0.863
Veery (*Catharus fuscescens*)	Woodland	0.041 (0.007)	0.001 (<0.001)	0.206 (0.069)	0.551
Wood Thrush (*Hylocichla mustelina*)	Woodland	NE	0.141 (0.026)	0.514 (0.024)	NE
Brown Thrasher (*Toxostoma rufum*)	Scrub-shrub	0.749 (0.038)	0.139 (0.070)	0.615 (0.089)	0.525
Prothonotary Warbler (*Protonotaria citrea*)	Other	0.111 (0.009)	NE	0.232 (0.050)	0.696
Kentucky Warbler (*Geothlypis formosa*)	Woodland	0.001 (<0.001)	0.101 (0.044)	0.212 (0.051)	0.795
Common Yellowthroat (*Geothlypis trichas*)	Grassland	0.995 (0.004)	NE	0.935 (0.006)	0.640
Cerulean Warbler (*Setophaga cerulea*)	Woodland	0.179 (0.102)	0.129 (0.129)	0.685 (0.053)	0.722
Field Sparrow (*Spizella pusilla*)	Scrub-shrub	0.750 (0.028)	0.217 (0.052)	0.752 (0.016)	0.592
Grasshopper Sparrow (*Ammodramus savannarum*)	Grassland	0.346 (0.031)	0.091 (0.034)	0.581 (0.031)	0.661
Henslow's Sparrow (*Ammodramus henslowii*)	Grassland	0.043 (0.012)	0.018 (0.009)	0.628 (0.051)	0.589
Dickcissel (*Spiza americana*)	Grassland	0.492 (0.015)	0.346 (0.011)	0.457 (0.109)	0.766
Bobolink (*Dolichonyx oryzivorus*)	Grassland	0.396 (0.039)	0.183 (0.062)	0.921 (0.045)	0.848
Eastern Meadowlark (*Sturnella magna*)	Grassland	0.458 (0.011)	0.238 (0.015)	0.782 (0.053)	0.713
Western Meadowlark (*Sturnella neglecta*)	Grassland	0.126 (0.002)	0.111 (0.002)	0.406 (0.032)	0.924
Baltimore Oriole (*Icterus galbula*)	Woodland	0.948 (0.030)	0.999 (<0.001)	0.509 (0.022)	0.668

“NE” denotes parameter not estimated.

Using estimates of effect size on covariates from the best model for each species, we predicted cell-specific values of ψ and γ across all of Iowa for each species. To develop a predictive map of both parameters for each species, we first established a 1000 m point grid across the entire state resulting in a total sample of 145,729 points across Iowa. We used these points as a basis for assessing landscape-level habitat characteristics of interest across all of Iowa. Repeating the process described above for our sampled sites, we placed a buffer around each point, clipped the 2009 Iowa Landcover file to each buffer, and estimated the above-mentioned landscape-level habitat characteristics for each of the three land-use classifications. This process was completed for a 200 m, 500 m, and 1000 m radius around each point. Once we successfully estimated landscape-level habitat characteristics for each of the 145,729 points across Iowa, we then developed predictive models for each parameter for every species using the linear coefficients of the covariate effects on the respective parameter from the best model. We calculated a value for both Ψ and γ for each point in the point grid by taking the logit transformation of the product of the linear coefficient of the covariate or covariates on ψ in the best model and the value for the covariate at the respective point.

### Model predictions

To create the map, we interpolated values of ψ and γ between points in our point grid using the kriging tool in ArcGIS [Spatial Analyst Tools, Interpolation, Kriging; 29]. This process involved generating a raster surface from points by interpolating values between points based on values for established points within a specified search distance (m). Within the kriging tool, we specified a spherical semivariogram model, set our output cell size to match the radius of the landscape included in the best robust design occupancy model for the particular species (200 m, 500 m, or 1000 m), and set our maximum search distance to 1000 m so the interpolation would only consider adjacent points in the point grid. Because the size of our cells for prediction were 1000 m^2^, we simply used raster algebra to multiply the covariate value of each individual cell by the effect size of that covariate. Kriging was only used to interpolate among prediction cells for the 200 m and 500 m scales.

### Model validation

We evaluated our models using the area under the receiver operating characteristic curve (AUC), a threshold-independent procedure that compares the distributions of correctly and incorrectly classified predictions over a wide range of threshold levels [41]. An average AUC score of 0.5 represents a prediction of random choice whereas an average AUC score of 1.0 is a perfect prediction [42]. We used survey year 2013 as our test data set and survey years 2006–2012 and 2014 as our training data set [43]. We selected survey year 2013 as our test data set, which represented approximately 20% of the total number of properties surveyed, because properties surveyed in 2013 were better representative of the spatial variability of habitat across Iowa. This approach is used frequently in the literature for evaluating performance of logistic regression and occupancy models for predicting occupancy probability [41,44–46]. We considered models useful if the respective AUC was > 0.70 [47]. We did not evaluate models for probability of colonization due to our lack of data for doing so. Evaluating models for probability of colonization would require multiple sites with repeated visits in our test data set (i.e. survey year 2013), of which we only had five. We predicted probability of colonization for each species because such values are important for conservation planning. However, we suggest readers use caution when interpreting these values since they are not validated using an independent data set.

### Cumulative maps

Once we obtained predictive maps for each species, we created additional cumulative maps that predicted species richness and colonization for all species combined. We also created cumulative maps of predicted species richness and colonization for species with predictive models considered useful (AUC > 0.70) within each of the grassland, woodland, and scrub-shrub species groups. Cumulative maps were created by calculating the sum of the respective probabilities for all species considered for each map [48]. We did not create cumulative maps for species in the “other” group because all species within that group either did not have all parameters estimated or did not have predictive models considered useful.

## Results

We surveyed a total of 292 properties across Iowa from 2006–2014 of which 272 were surveyed only one year and 20 were surveyed in more than one year (Fig 1). Detections of individual species ranged from 4–1354 (mean = 261) with common nighthawk (*Chordeiles minor*) detected on the fewest occasions and common yellowthroat (*Geothlypis trichas*) detected on the most occasions.

### Robust design occupancy models

For most species, the best predictors of occupancy and colonization were at the 500 m spatial scale (Table 3). Covariates at the 500 m spatial scale were included in the best model for occupancy for 21 species and in the best model for colonization for 19 species. Covariates at the 1000 m spatial scale were also important predictors of occupancy and colonization for eight species and 11 species, respectively. Only one species responded to covariates at the 200 m spatial scale for occupancy (upland sandpiper [*Bartramia longicauda*]) and colonization (northern harrier [*Circus cyaneus*]).

**Table 3:** List of best models for each species and the effect size (Psi, Gam, p) and 95% confidence interval (95% CI) for the covariate on each parameter in the model.

Species	Model	Psi	95% CI	Gam	95% CI	p	95% CI
Trumpeter Swan	Psi(~Ag1KPLND)Gam(~Wtr500PLND)p(~1)	**0.059**	**(0.051, 0.067)**	**0.129**	**(0.103, 0.154)**		
Northern Bobwhite	Psi(~Grs500PLND)Gam(~Grs500PD)p(~Wind)	**0.055**	**(0.053, 0.057)**	**0.004**	**(0.004, 0.004)**	**-0.176**	**(-0.298, -0.055)**
American Bittern	Psi(~Wtl1kPLND)Gam(~1)p(~Cld)	**0.252**	**(0.052, 0.453)**			**0.014**	**(0.001, 0.027)**
Bald Eagle	Psi(~Wtr1kED)Gam(~Ag500LPI)p(~1)	0.022	(-0.001, 0.046)	**0.132**	**(0.032, 0.232)**		
Northern Harrier	Psi(~Wod500PLND)Gam(~Wod200LPI)p(~Temp)	**-0.512**	**(-0.528, -0.495)**	**-0.039**	**(-0.043, -0.035)**	**-0.064**	**(-0.087, -0.040)**
Red-shouldered Hawk	Psi(~Wod1KPLND)Gam(~Ag1KPD)p(~Cld)	**0.034**	**(0.028, 0.040)**	**0.002**	**(0.002, 0.002)**	-0.008	(-0.016, 0.000)
Upland Sandpiper	Psi(~Grs200PLND * Wod200PLND)Gam(~Ag1kLPI)p(~Wind)	0.077	(-0.017, 0.171)	**0.071**	**(0.008, 0.135)**	-0.184	(-0.396, 0.027)
Yellow-billed Cuckoo	Psi(~Wod500PLND)Gam(~Wod1kLPI)p(~Wind)	**0.060**	**(0.050, 0.069)**	**0.042**	**(0.025, 0.059)**	**-0.076**	**(-0.140, -0.012)**
Black-billed Cuckoo	Psi(~Wod1KPLND)Gam(~Ag500PLND)p(~1)	**0.146**	**(0.033, 0.258)**	**-0.287**	**(-0.481, -0.092)**		
Belted Kingfisher	Psi(~Wtr500ED)Gam(~Wtl500PLND)p(~1)	**0.010**	**(0.002, 0.019)**	-0.114	(-0.267, 0.040)		
Red-headed Woodpecker	Psi(~Wod500PLND)Gam(~Ag500PD)p(~Cld)	**0.017**	**(0.009, 0.025)**	**-0.002**	**(-0.004, 0.000)**	**-0.004**	**(-0.008, -0.001)**
Northern Flicker	Psi(~Wod500ED)Gam(~Wod500PLND * Wod500LPI)p(~Wind)	**0.009**	**(0.005, 0.012)**	**0.032**	**(0.002, 0.061)**	**-0.119**	**(-0.164, -0.074)**
Eastern Wood-Pewee	Psi(~Wod500PLND)Gam(~Wod1KPLND)p(~Wind)	**0.128**	**(0.125, 0.131)**	**0.071**	**(0.069, 0.072)**	**-0.110**	**(-0.168, -0.052)**
American Kestrel	Psi(~Grs500LPI)Gam(~Grs500LPI)p(~1)	**0.123**	**(0.119, 0.126)**	**0.922**	**(0.320, 1.525)**		
Acadian Flycatcher	Psi(~Wod500PLND)Gam(~Wod500PLND)p(~Wind)	**0.092**	**(0.090, 0.094)**	**0.07**	**(0.068, 0.072)**	0.086	(-0.020, 0.192)
Eastern Kingbird	Psi(~Wod500LPI)Gam(~Ag1kLPI)p(~1)	**-0.050**	**(-0.051, -0.050)**	**-0.145**	**(-0.150, -0.141)**		
Bell's Vireo	Psi(~Wod1KPD)Gam(~Ag500PD)p(~Wind)	**0.006**	**(0.006, 0.006)**	**0.003**	**(0.003, 0.003)**	**0.351**	**(0.049, 0.652)**
Bank Swallow	Psi(~Ag1KPLND)Gam(~Wtl1kPLND)p(~1)	**0.030**	**(0.024, 0.036)**	**-0.063**	**(-0.110, -0.016)**		
Sedge Wren	Psi(~Wtl500PLND * Grs500PLND)Gam(~Wtl500PLND * Grs500PLND)p(~1)	**0.008**	**(0.008, 0.009)**	**0.008**	**(0.007, 0.008)**		
Veery	Psi(~Wod1kED)Gam(~Ag500LPI)p(~Wind)	**0.005**	**(0.004, 0.006)**	**-0.578**	**(-0.655, -0.501)**	0.179	(-0.005, 0.362)
Wood Thrush	Psi(~Wod500LPI)Gam(~Wod500PLND)p(~Wind)	-2.065	(-4.839, 0.710)	**0.063**	**(0.047, 0.078)**		
Brown Thrasher	Psi(~Grs500ED * Wod500ED)Gam(~Grs1KPD)p(~Temp)	**0.003**	**(0.001, 0.005)**			**-0.013**	**(-0.025, -0.002)**
Prothonotary Warbler	Psi(~Wod500PD)Gam(~Wod500PLND * Wtl500PD)p(~1)	**-0.017**	**(-0.018, -0.015)**				
Kentucky Warbler	Psi(~Wod500PD)Gam(~Wod500PLND)p(~Wind)	-0.073	(-0.195, 0.049)	**0.061**	**(0.012, 0.109)**	**0.162**	**(0.023, 0.301)**
Common Yellowthroat	Psi(~Wtl500PLND * Grs500PLND)Gam(~Wtl1kLPI)p(~1)	-0.026	(-0.141, 0.090)				
Cerulean Warbler	Psi(~Wod1KPD)Gam(~Wod1KPD)p(~1)	**-0.050**	**(-0.086, -0.014)**	-0.052	(-0.129, 0.024)		
Field Sparrow	Psi(~Wod500PD)Gam(~Wod500PD)p(~Wind)	**0.008**	**(0.004, 0.011)**	0.003	(-0.001, 0.007)	**-0.094**	**(-0.140, -0.049)**
Grasshopper Sparrow	Psi(~Grs500PLND)Gam(~Wod1KPLND)p(~Wind)	**0.048**	**(0.032, 0.064)**	**-0.041**	**(-0.078, -0.003)**	-0.066	(-0.134, 0.003)
Henslow's Sparrow	Psi(~Grs1KPLND)Gam(~Grs1KPLND)p(~1)	**0.060**	**(0.031, 0.088)**	**0.090**	**(0.029, 0.151)**		
Dickcissel	Psi(~Wod500PLND)Gam(~Wod500PLND)p(~Temp)	**-0.064**	**(-0.067, -0.062)**	**-0.042**	**(-0.043, -0.040)**	**0.014**	**(0.001, 0.028)**
Bobolink	Psi(~Grs500PLND)Gam(~Grs500LPI)p(~Temp)	**0.103**	**(0.077, 0.129)**	**0.115**	**(0.030, 0.200)**	**-0.036**	**(-0.055, -0.016)**
Eastern Meadowlark	Psi(~Wod500LPI)Gam(~Ag500LPI)p(~Temp)	**-0.045**	**(-0.046, -0.043)**	**0.079**	**(0.065, 0.093)**	**-0.021**	**(-0.031, -0.011)**
Western Meadowlark	Psi(~Wod500PLND)Gam(~Grs500LPI)p(~Wind)	**-0.058**	**(-0.058, -0.057)**	**0.052**	**(0.050, 0.053)**	**0.087**	**(0.021, 0.152)**
Baltimore Oriole	Psi(~Wod500ED)Gam(~Wod500PLND)p(~Wind)	**0.013**	**(0.007, 0.020)**	0.281	(-0.058, 0.620)	**0.110**	**(0.062, 0.158)**

Covariates modeled on Psi and Gam are a combination of the following abbreviations: “Ag” represents agriculture, “Wtr” represents water, “Wtl” represents wetland, “Grs” represents grassland, “Wod” represents woodland, “200” represents the 200 m spatial scale, “500” represents the 500 m spatial scale, “1k” represents the 1000 m spatial scale, “PLND” represents percentage of the landscape, “PD” represents patch density, “ED” represents edge density, and “LPI” represents largest patch index. Therefore, as an example, “Ag1kPLND” represents the percentage of the landscape in agriculture at the 1000 m spatial scale. Covariates modeled on p are as follows: “Wind” represents wind speed (km/h), “Cloud” represents cloud cover (%), “Temp” represents temperature (°C), and “1” represents a constant effect. *Bold text indicates a significant effect (confidence interval did not include zero)*.

For woodland species, the most important predictor (covariate included in best model for most species) of occupancy and colonization was percentage of the landscape in woodland at either the 500 m or 1000 m spatial scales (Table 3). Occupancy of most grassland species was either positively correlated with the percentage of the landscape in grassland at either the 200 m, 500 m, or 1000 m spatial scales or negatively correlated with various characteristics of woodland on the landscape (Table 3). Colonization of grassland species was not frequently correlated with any one covariate and included a negative correlation with woodland characteristics, a mix of positive and negative correlations with agriculture characteristics, and positive correlations with grassland characteristics on the landscape. As expected, occupancy of most scrub-shrub species was positively associated with both grassland and woodland characteristics that would suggest the use of edge habitat such as patch density of both grassland, edge density of both grassland and woodland, and percentage of the landscape in both grassland and woodland, most of which at the 500 m spatial scale (Table 3). Colonization of scrub-shrub species showed similar correlations. However, colonization of two scrub-shrub species (black-billed cuckoo [*Coccyzus erythropthalmus*] and Bell’s vireo [*Vireo bellii*]) were negatively associated with the percentage of the landscape in agriculture at the 500 m spatial scale and positively associated with the patch density of agriculture at the 500 m spatial scale, respectively (Table 3). For all other species, occupancy was positively correlated with a variety of characteristics including edge density of water at the 500 m spatial scale, percentage of the landscape in agriculture at the 1000 m spatial scale, and percentage of the landscape in wetlands at the 1000 m spatial scale. Colonization was not estimated, likely due to lack of opportunity in the data, or exhibited a non-significant correlation with one or more characteristics for most other species. However, colonization was positively correlated with the percentage of the landscape in water at the 500 m spatial scale for trumpeter swan (*Cygnus buccinator*) and the largest patch index of agriculture at the 500 m spatial scale for bald eagle (*Haliaeetus leucocephalus*), and was negatively correlated with the percentage of the landscape in wetlands at the 1000 m spatial scale for bank swallow (*Riparia riparia*; Table 3). Wind speed was the most frequent covariate affecting detection probability, appearing as an important covariate in the best model for 14 of our 34 species (Table 3). A constant effect on detection probability was also important, appearing in the best model for 12 of 34 species (Table 3). Temperature and cloud cover were important covariates on detection probability for five and three species, respectively.

Occupancy probability ranged from 0.030 to 0.995 for all species (Table 2), with the lowest occupancy probability estimated for acadian flycatcher (*Empidonax virescens*) and the highest occupancy probability estimated for common yellowthroat. Occupancy probability was estimated as zero or was not estimated for eight species. Occupancy probabilities were generally higher for scrub-shrub species (mean = 0.477) than for grassland (mean = 0.354), woodland (mean = 0.404), and all other species (mean = 0.247). Colonization probability ranged from 0.020 for Henslow’s sparrow (*Ammodramus henslowii*) to 0.481 for eastern wood-pewee (*Contopus virens*). Colonization probability was generally greater for grassland species (mean = 0.315) than for scrub-shrub (mean = 0.171), woodland (mean = 0.241), and all other species (mean = 0.270). Colonization probability was not estimated for nine species. The lowest detection probability, 0.055, was estimated for American kestrel (*Falco sparverius*) and the highest detection probability, 0.935, for common yellowthroat. Detection probability was generally greater for grassland species (mean = 0.592) than for scrub-shrub (mean = 0.473), woodland (mean = 0.508), and all other species (mean = 0.186). Detection probability was not estimated for four species.

### Model validation

Predictive models for occupancy were considered useful (AUC > 0.70) for 17 of 31 species for which models were evaluated. AUC values for occupancy probability ranged from 0.525 for brown thrasher (*Toxostoma rufum*) to 0.924 for western meadowlark (*Sturnella neglecta*). We did not evaluate models for eight species for which occupancy probability was not estimated.

## Discussion

**Fig 2:**
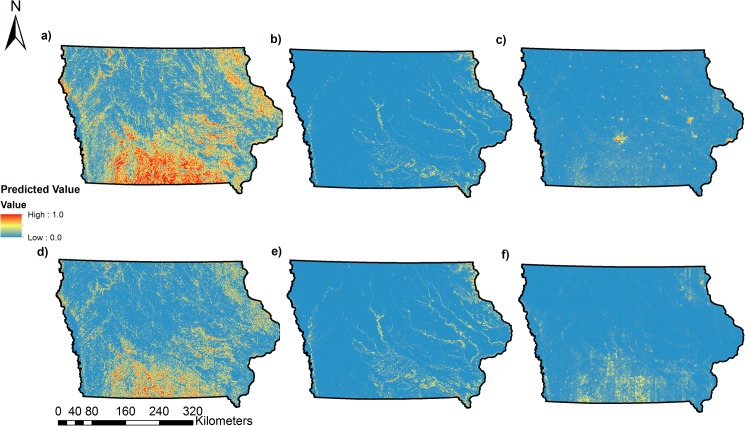
Predicted probability of occupancy and colonization for three bird Species of Greatest Conservation Need (SGCN) in Iowa using the covariates on Psi and Gamma from the best model for each species. Maps display values for one grassland species (Bobolink [*Dolichonyx oryzivorus*]), one woodland species (Acadian Flycatcher [*Empidonax virescens*]), and one scrub-shrub species (Bell’s Vireo [*Vireo bellii*]), all of which had predicted models considered useful (AUC > 0.70). (a) Predicted probability of occupancy for Bobolink, (b) Predicted probability of occupancy for Acadian Flycatcher, (c) Predicted probability of occupancy for Bell’s Vireo, (d) Predicted probability of colonization for Bobolink, (e) Predicted probability of colonization for Acadian Flycatcher, (f) Predicted probability of colonization for Bell’s Vireo.

**Fig 3:**
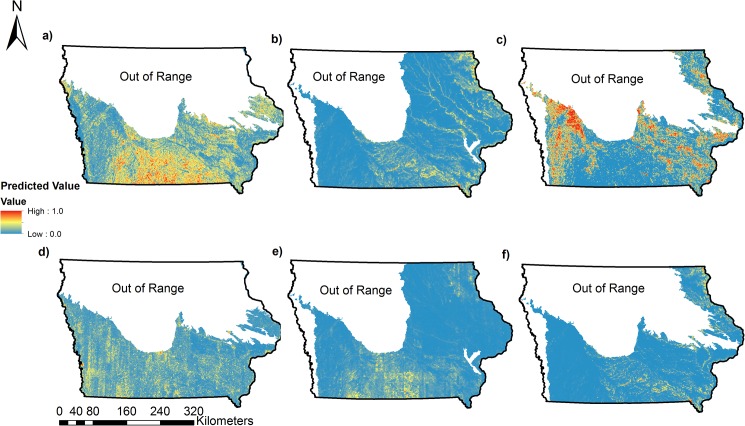
Predicted probability of occupancy and colonization for three range-restricted bird Species of Greatest Conservation Need (SGCN) in Iowa using the covariates on Psi and Gamma from the best model for each species. Predictive models for all species displayed were considered useful (AUC > 0.70). (a) Predicted probability of occupancy for Northern Bobwhite (*Colinus virginianus*), (b) Predicted probability of occupancy for Red-shouldered Hawk (*Buteo lineatus*), (c) Predicted probability of occupancy for Kentucky Warbler (*Geothlypis formosa*), (d) Predicted probability of colonization for Northern Bobwhite, (e) Predicted probability of colonization for Red-shouldered Hawk, (f) Predicted probability of colonization for Kentucky Warbler.

**Fig 4:**
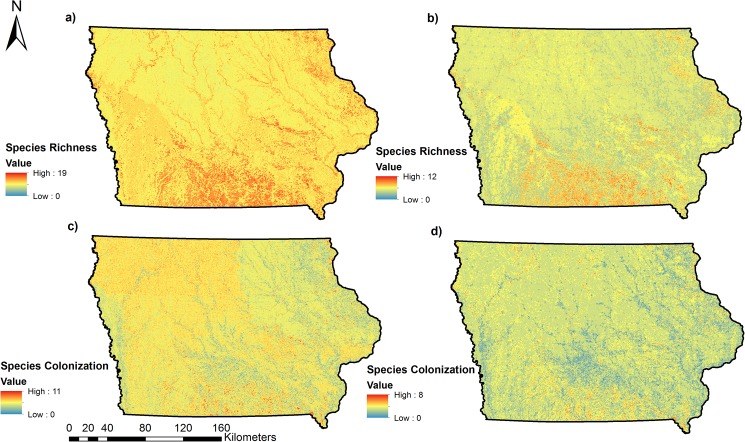
Predicted species richness and species colonization for all bird Species of Greatest Conservation Need (SGCN) included in this study in Iowa. Estimates were combined by calculating the sum of all estimated values of Psi and Gamma from the best model for each species. We combined estimates for all species included in the study for both Psi (a) and Gamma (c). We also combined estimates for those species with predictive models that were considered useful (AUC > 0.70) for both Psi (b) and Gamma (d).

**Fig 5:**
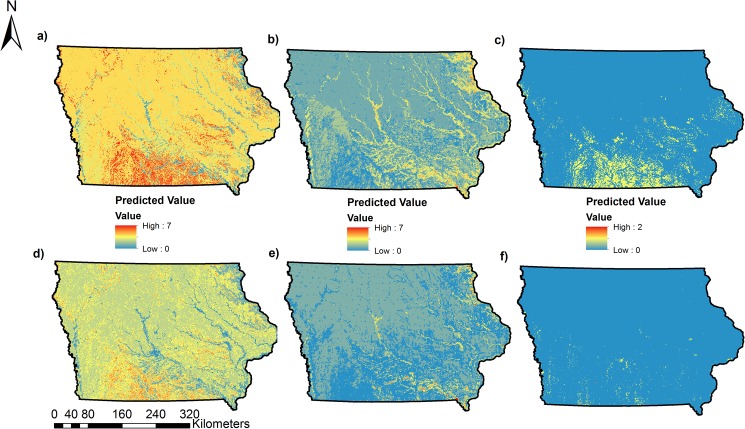
Predicted species richness and colonization for grassland, woodland, and scrub-shrub bird Species of Greatest Conservation Need (SGCN) in Iowa. Estimates were combined by calculating the sum of all estimated values of Psi and Gamma from the best model for each species with predictive models that were considered useful (AUC > 0.70) within each group. (a) Predicted species richness for grassland bird SGCN, (b) Predicted species richness for woodland bird SGCN, (c) Predicted species richness of scrub-shrub bird SGCN, (d) Predicted species colonization for grassland bird SGCN, (e) Predicted species colonization for woodland bird SGCN, (f) Predicted species colonization for scrub-shrub SGCN.

Our approach uses data from a long-term monitoring program to assess landscape habitat associations and predict occupancy and colonization as a function of landscape variables obtained from high-resolution (3 m) landcover data (Fig 2, Fig 3, Fig 4, Fig 5). For 17 of 31 species, our predictive models for occupancy were considered, thus illustrating the utility of our approach in predicting distributions for some species of conservation concern. Using models that incorporate imperfect detection minimizes the possibility of underestimating occupancy and colonization and thus the extent of the potential distribution and colonization of each species [49]. Our study is one of few to predict the probability of colonization for multiple species [17], a parameter that can be very useful to managers for targeting habitat restoration efforts in areas not currently occupied by a species of interest.

### Landscape habitat associations

Percentage of the landscape in a particular habitat class was the most important characteristic predicting occupancy of most of our study species. The probability of occupancy for most woodland, grassland, and scrub-shrub species was positively correlated with percentage of the landscape in woodland, grassland, or a combination of both, respectively. One of the fundamental requirements of adequate habitat is sufficient space for an animal to move, locate a mate, avoid potential predators or aggressive interactions with conspecifics, and obtain food and water, all of which are critical to its well-being [50]. Lack of sufficient space can reduce the survival of an individual animal, which can decrease the carrying capacity of an area and ultimately result in population declines of a species [50]. The importance of space, or the amount of habitat on the landscape, has been demonstrated in the literature. For example, a previous study [33] demonstrated that species richness of woodland birds decreases drastically as cover of trees drops below 10% on the landscape. For grassland birds, studies have also found the abundance of grassland habitat on the landscape influences probability of occupancy and species richness of grassland bird species at a particular area [32,51–53]. Our study not only established percentage of habitat on the landscape as an important predictor of bird probability of occupancy, but also predicted areas of both high and low probabilities of occupancy for species based on this landscape characteristic. This study is important to land managers interested in restoring and managing habitat for birds of conservation concern in two ways: 1) areas of high species occupancy inform managers where to focus habitat management efforts, particularly if the species is confirmed at the site, and 2) areas of low occupancy inform managers where to focus habitat restoration and land acquisition efforts to increase the suitability of the area for the species of interest. This study is also important for future research because it illustrates areas to focus surveys, especially for species which lack data on population trends, and to conduct on-site evaluations of species habitat associations.

Although other studies have evaluated colonization of habitat patches by various bird species [54–56], no study to our knowledge has used this information to predict probability of colonization of multiple bird species on the landscape. Patch isolation is frequently cited as a significant influence of patch colonization by birds [55,56]. Additionally, patch size is not only an important influence on colonization but also influences the persistence of a species at a particular patch [54]. Largest patch index was an important predictor of probability of colonization for some woodland and grassland species in our study. However, the amount of habitat on the landscape (i.e., PLAND) was of greater importance for colonization probability of both grassland and woodland species. Percentage of the landscape in woodland at various spatial scales was positively correlated with probability of colonization for five woodland species and negatively correlated with probability of colonization for two grassland species. Percentage of the landscape in grassland at various spatial scales was positively correlated with probability of colonization for two grassland species. These results suggest that, although patch size is an important predictor of probability of colonization for some birds, the amount of habitat on the landscape is of greater importance to probability of colonization particularly on an intensively modified landscape such as Iowa. Greater than 99% of the Iowa landscape has been converted to agriculture, and the influence of this drastic change on probability of colonization is evident in our results. Probability of colonization for eight species was significantly correlated, either positively or negatively, with either the amount of agriculture on the landscape of the size of agriculture patches on the landscape. This result should be interpreted with caution because it is possible that the effect of these agriculture variables on our parameters is a result of the high amount and lack of variation of agriculture on the landscape. Conversely, this result could suggest that some species such as upland sandpiper are successfully colonize areas with increased agriculture on the landscape whereas other species such as black-billed cuckoo and eastern kingbird will only colonize areas with less agriculture on the landscape. Nonetheless, the ability to predict probability of colonization on any landscape is critical to prioritizing areas of conservation actions for birds.

There are caveats to our approach that need to be considered. First, we did not consider annual variation when estimating both occupancy and colonization probabilities, both of which are expected to vary annually [17]. We modeled both occupancy and colonization probabilities as a function of landscape covariates obtained for a single year of landcover data. Because our landscape covariates were constant across all years of monitoring data, we did not expect occupancy and colonization probabilities to vary annually. Although we acknowledge the likelihood of the landscape changing during the duration of our study, obtaining high-resolution landcover data on an annual basis is not feasible. Additionally, we did not incorporate on-site habitat characteristics in our models. Several studies speak to the value of incorporating both landscape- and local-level variables in analyses of habitat associations [57,58]. However, these studies were typically focused on small geographic areas and not conducted at a statewide level. Furthermore, there are currently no sources of on-site habitat data for Iowa in its entirety, which would render prediction of occupancy and colonization based on these characteristics impossible. We were unable to validate predicted values for probability of colonization due to lack of data in our test data set. However, outlining the approach for predicting probability of colonization is important for repeatability of our study and applicability to other data sets. Lastly, we were unable to develop useful predictive models for 14 of our 31 study species. This could be due to lack of sufficient data or because we didn’t include appropriate covariates for these individual species in our models, which is related to other caveats mentioned above. Although predictive models for these species were not useful for management purposes, it didn’t necessarily mean that the model isn’t valid [59]. Collection of additional data could help improve these models for future use. Despite these caveats, we contend that our results provide valuable information to scientists and land managers that informs future research and management efforts on bird species of conservation concern.

### Value of predictive models

Predictive species occupancy and colonization models have high value for conservation planning. Such models provide valuable information for developing strategies to prioritize conservation action, an effort that will continue to be critical in conserving biodiversity throughout the world [13]. Predictive species occupancy models are important for planning wildlife reserve networks, suggesting benefits to (1) the land manager by providing information to focus conservation efforts, and (2) to the species by affecting habitat management and restoration in areas of greatest potential use [15]. In a period of reduced funding for conservation, unbiased knowledge of species occurrence is especially important in effective conservation spending [60].

### Conclusions

Reduced funding is resulting in increased pressure for state and provincial fish and wildlife agencies to focus spending in areas of high conservation potential. Despite its success in preventing endangered species listings in several states since its inception in 2001, the State and Tribal Wildlife Grants Program was never fully funded and has experienced a 35% decline in funds allocation since 2010 [14]. At yet a smaller scale, counties and municipalities cite a lack of staff and funding for conservation planning in their jurisdictions [61]. The lack of staff and funding for conservation makes the prioritization of areas through predictive species occupancy models very important. Reduced funding aside, the lack of reliable scientific information on distributions of species of conservation concern is making implementation of conservation plans difficult for state, provincial, and local governments [61,62]. Our study provides a practical framework for predicting species occupancy and colonization from a long-term monitoring data set which builds off methods provided in other studies [17,49] and can be applied to other areas where data are available. This approach produces predictive maps which requires little interpolation of occupancy and colonization values among points with a large degree of spatial separation. Our approach can be utilized with data from other state or regional long-term monitoring programs as well as other landscape-scale habitat data (e.g., National Land Cover Database [NLCD] or other state landcover data sets). As threats to biodiversity continue to increase, predictive modeling for conservation planning will become increasingly important in the efforts to preserve biodiversity into the future.
